# Application of Stress and Anxiety to Viral Epidemics-6 Items (SAVE-6) to Public Workers for Measuring Their Anxiety Response During the COVID-19 Pandemic

**DOI:** 10.3389/fpsyt.2021.701543

**Published:** 2021-10-06

**Authors:** C. Hyung Keun Park, Gawon Ju, Kikyoung Yi, Sangha Lee, Sooyeon Suh, Seockhoon Chung

**Affiliations:** ^1^Department of Psychiatry, Asan Medical Center, University of Ulsan College of Medicine, Seoul, South Korea; ^2^Department of Psychiatry, Chungbuk National University Hospital, Cheongju, South Korea; ^3^Yong-In Mental Hospital, Yongin, South Korea; ^4^Department of Psychiatry, Ajou University School of Medicine, Suwon, South Korea; ^5^Department of Psychology, Sungshin Women's University, Seoul, South Korea

**Keywords:** public workers, COVID-19, SAVE-9, anxiety, stress

## Abstract

**Objective:** This study aimed to compare the adaptability of the adapted version of Stress and Anxiety to Viral Epidemics-9 (SAVE-9) for public workers and the SAVE-6 scale and to validate them among public workers who are on the frontline of the coronavirus disease 2019 pandemic.

**Methods:** A total of 300 public workers responded to the anonymous online survey during April 1–12, 2021. Principal component analysis was conducted with varimax rotation to explore the factor structure of this scale. Confirmatory factor analysis was also used to explore construct validity. Spearman correlation analysis of the scale with the Generalized Anxiety Disorder-7 (GAD-7) and the Patient Health Questionnaire-9 (PHQ-9) was performed to explore the convergent validity. The cut-off score in accordance with the mild degree of generalized anxiety symptoms (GAD-7 score of 5) was defined using the receiver operating characteristic (ROC) analysis.

**Findings:** The single-structure model of each scale (the adapted version of SAVE-9 and SAVE-6) was adopted based on the results of the parallel analysis. Because SAVE-6 showed good construct validity, but the adapted version of SAVE-9 did not, we adopted to apply the SAVE-6 scale to assess the anxiety response of public workers in response to the viral epidemic. SAVE-6 showed good internal consistency (Cronbach's alpha = 0.817; McDonald's Omega = 0.818) and good convergent validity with GAD-7 (rho = 0.417, *p* < 0.001) and PHQ-9 (rho = 0.317, *p* < 0.001) scale scores. The appropriate cut-off score for SAVE-6 was determined to be ≥ 16.

**Conclusion:** The SAVE-6 scale, as compared to the public workers' version of SAVE-9, is a reliable and valid rating scale to assess the work-related stress and anxiety of public workers due to the viral epidemic.

## Introduction

The outbreak of the coronavirus disease 2019 (COVID-19) first occurred in Wuhan, China, in December 2020. It is caused by the novel coronavirus (SARS-CoV-2), which has infected people worldwide. As of August 8, 2021, 203,394,686 confirmed cases and 4,306,521 deaths due to COVID-19 have been reported (1). Since the first reported case in Korea in January 2020, 212,438 cases and 2,125 deaths have been confirmed as of August 8, 2021 (2). As the pandemic worsens, the world is threatened not only by the disease but also by psychological issues such as depression and anxiety (3), making the assessment of mental health essential.

Like many other work employees, public workers face various challenges during the pandemic. They are pushed to the forefront of the struggle. They cope with a rapidly changing situation by making policies that can have a national impact. In these unprecedented times, moreover, the public workers are expected to have an attitude of self-sacrifice. As presented in the public brief recently published by the United Nations Department of Economic and Social Affairs, “providing service before self: courage and humanness in practice” is one of the important roles of the public workers nowadays (4). Besides, public health personnel directly involved in COVID-19-related fields perform stressful tasks such as developing and implementing regulations on wearing masks and social distancing that have enormous social consequences or even spark intense debate (5, 6). Because the current situation can impose unique psychological issues on public workers, they may have mental health concerns that are specific to their group.

During this pandemic, healthcare workers have suffered from work-related stress, anxiety, and depression while working to manage confirmed and high-risk cases. They are easily exposed to infectious diseases, and, in particular, those in direct contact with patients have the highest level of risk of infection (7). During a pandemic of an infectious disease such as COVID-19, the risk can result in work-related stress and burnout (8). Moreover, a number of healthcare workers experience psychiatric problems; over 20% report symptoms of depression and anxiety, and ~40% report sleep-related problems (9). Similarly, public workers suffer from severe burnout and psychological problems. In fact, public workers perform duties similar to those of healthcare workers because the former also interact with patients who may be infected with the virus, and they participate in transporting them in addition to conducting administrative duties related to COVID-19. Moreover, they may experience anxiety that they might pass the virus on to their families or people around them. Similar to healthcare workers experiencing the risk of infection, the risk of transmission, and work-related stress while treating patients, public workers are placed in a similar situation due to various duties while serving citizens. Thus, it is important to evaluate stress experienced by public workers and establish a support system. However, there is a paucity of studies on how to measure these workers' stress.

There are few studies on the mental health of public workers during the COVID-19 pandemic (10). Furthermore, viral epidemic-specific anxiety measures for public workers have not been developed. Recently, to assess the anxiety response of healthcare workers specifically in relation to the viral epidemic, we developed the Stress and Anxiety to Viral Epidemics-9 items (SAVE-9) scale to measure healthcare workers' work-related stress and anxiety specifically in regard to the COVID-19 pandemic (11). The SAVE-9 scale was clustered into two factors: Factor I, “Anxiety regarding the epidemic” [items 1, 2, 3, 4, 5, and 8, namely SAVE-6 (12)], and Factor II, “Work-related stress associated with the epidemic” (items 6, 7, and 9). We observed that the SAVE-9 scale was a reliable (Cronbach's alpha = 0.795) and valid (with the Generalized Anxiety Disorder-I items scale, GAD-7, rho = 0.51, p < 0.001) rating scale. The appropriate cut-off point was ≥ 22 (sensitivity = 0.67, specificity = 0.68) in accordance with a GAD-7 score of 5, which indicates a mild degree of generalized anxiety. Considering the pressures that public workers can receive in relation to their work under the pandemic, it will be necessary to assess the public workers' anxiety or work-related stress. Though we can utilize another rating scale that is widely used but not specific to the viral epidemic, developing anxiety measures for public workers specifically relating to the viral epidemic would be valuable. Because the SAVE-9 scale was originally developed for healthcare workers, in this study, we attempted to adapt this scale into a public workers' version that can be applied to assess public workers' work-related stress and anxiety response to the viral epidemic, specifically. In this study, we aimed to explore the structural validity and applicability of the adapted public worker version of the SAVE-9 scale. Moreover, we also applied the SAVE-6 scale (12), derived from the SAVE-9 scale for application to the general population, to the same sample to explore which of the two—the adapted SAVE-9 scale or the adapted SAVE-6 scale—is more applicable to measure the anxiety response of public workers to the viral epidemic.

## Methods

### Participants and Procedure

This study was conducted via an online survey with the professional survey company, EMBRAIN^1^, during April 1–12, 2021. A total of 300 public workers were enrolled through the survey system who voluntarily responded to the questions. This survey was anonymous, and no personal information was gathered. The study protocol was approved by the Institutional Review Board of the Asan Medical Center (2021-0448), and written informed consent for participation was waived. Because sample size is typically determined by the rule that the ideal ratio of respondents to items is 10:1 (13), at least 90 participants were needed to validate the scale in this study. However, we planned to gather 300 public workers to develop the scale based on the suggestion that a range of 200–300 is appropriate for factor analysis (14, 15).

### Symptom Assessment

#### (1) Stress and Anxiety to Viral Epidemics-9 items (SAVE-9) adapted for public workers

The original SAVE-9 scale was developed to assess healthcare workers' work-related stress and anxiety due to the viral pandemic (11) The original SAVE-9 scale was clustered into two factors: Factor I, “Anxiety about the epidemic” (SAVE-6 (12), items 1, 2, 3, 4, 5, and 8), and Factor II, “Work-related stress associated with the epidemic” (items 6, 7, and 9). The Cronbach's alpha of the original SAVE-9 was 0.795 among a sample of healthcare workers, and the appropriate cut-off point was ≥ 22 (sensitivity = 0.67, specificity = 0.68) in accordance with an at least mild degree of generalized anxiety (GAD-7 score of 5). In this study, we adapted the original item 7, changing it from “After this experience, do you think you will avoid dealing with visitors with viral illnesses?” to “After this experience, do you think you will avoid dealing with clients with viral illnesses?” The items are rated on a 5-point Likert scale: 0 (never), 1 (rarely), 2 (sometimes), 3 (often), and 4 (always)^2^. In this study, we applied the adapted version of the SAVE-9 scale and the original SAVE-6 scale to compare their respective applicability to public workers. The SAVE-6 scale was derived from the original SAVE-9 scale to assess the anxiety response of the general population specifically regarding the viral epidemic (12). It was validated among the Korean and Lebanese general populations (16) as well as special populations of medical students (17) and cancer patients (18). Among the Korean population (12), the SAVE-6 scale showed good internal consistency (Cronbach's alpha = 0.815), and the appropriate cut-off point was ≥ 15 (sensitivity = 0.70, specificity = 0.60) in accordance with mild degree of generalized anxiety (GAD-7 score of 5).

#### (2) Generalized Anxiety Disorder-7 (GAD-7)

This scale is a self-administered questionnaire to assess the generalized anxiety of the people. It has seven items that are scored from 0 to 3 (0 = not at all to 3 = nearly every day), and the total score can range from 0 to 21. A higher score reflects a severe degree of generalized anxiety symptoms (19).

#### (3) Patient health questionnaire-9 (PHQ-9)

This scale is a self-administered questionnaire to assess one's depressive symptoms. It has nine items that are scored from 0 to 3 (0 = not at all to 3 = nearly every day), and the total score can range from 0 to 27. A higher score reflects severe degree of depressive symptoms (20).

### Statistical Analysis

We conducted principal component analysis (PCA) with varimax rotation and confirmatory factor analysis (CFA) to explore the factor structure of the adapted public worker version of the SAVE-9 and SAVE-6 scales. The normality assumption of each item was first checked using skewness and kurtosis for an acceptable limit of range ± 2 (21). The data suitability and sampling adequacy were assessed using Kaiser-Meyer-Olkin (KMO) value and Bartlett's test of sphericity. To determine the number of factors to retain for the adapted SAVE-9 and SAVE-6 scales, a scree test and parallel analysis (22–24), based on Minimum Rank Factor Analysis (MRFA) (25), with a 95 percentile threshold based on the polychoric correlations matrix was conducted using the FACTOR 10.10.03. program (25). We compared the explained real-data eigenvalues to the 95th percentile of random eigenvalues, and we decided where the real-data eigenvalues exceeded the 95th percentile of the random eigenvalues. A bootstrap (2,000 samples) maximum likelihood CFA was also conducted to explore the construct validity and applicability of the adapted SAVE-9 and SAVE-6 scales to assess the anxiety response of public workers in response to the viral epidemic. Satisfactory model fit was defined by a standardized root-mean-square residual (SRMR) value ≤ 0.05, root-mean-square-error of approximation (RMSEA) value ≤ 0.10, and comparative fit index (CFI) and Tucker Lewis index (TLI) values ≥ 0.90 (26, 27). Multi-group CFAs were conducted to explore whether the adapted SAVE-9 and SAVE-6 scales, if adopted, measure anxiety response the same way across genders (men vs. women), types of public workers (national government worker vs. local government worker), and the presence or absences of anxiety (GAD-7 ≥ 5 vs. GAD-7 < 5). We checked the reliability and internal consistency of the scale using Cronbach's alpha and McDonald's Omega. To explore the convergent validity, we performed Spearman correlation analysis of the adapted SAVE-9 or SAVE-6 scale score with PHQ-9 and GAD-7 scales, as the distributions of PHQ-9 and GAD-7 scores were not within the normal limit. Finally, we conducted receiver operating characteristic (ROC) analysis to define the appropriate cut-off score of the adapted SAVE-9 or SAVE-6 in accordance with the mild degree of generalized anxiety symptoms (GAD-7 score = 5). The SPSS version 21.0 (SPSS, Inc, Chicago, Illinois) and JASP version 0.14.1.0 software (JASP Team, Amsterdam, Netherlands) were also used for statistical analysis.

## Results

### Characteristics of the Participants

All 300 public workers participated in the online survey (Table 1). Among the participants, 60.0% were national government workers, and 40.0% were local government workers. Respondents were sampled from Seoul (*N* = 72, 24.0%), Pusan (*N* = 18, 7.0%), Daegu (*N* = 10, 3.3%), Daejeon (*N* = 17, 5.7%), Gwangju (*N* = 9, 3.0%), Incheon (*N* =15, 5.0%), Ulsan (*N* = 4, 1.3%), Sejong (*N* = 10, 3.3%), Gyeonggi Province (*N* = 51, 17.0%), Chungcheong Province (*N* = 18, 6.0%), Jeolla Province (*N* = 25, 8.3%), Gyeongsang Province (*n* = 28, 6.0%), Gangwon Province (*N* = 21, 7.0%), and Jeju Province (*N* = 2, 0.7%). The mean age was 38.3 ± 9.1 years, and the mean years of employment was 10.3 ± 8.8. With regards to questions related to COVID-19, 83 (27.7%) answered that they encountered confirmed COVID-19 cases, and 45 (15.0%) experienced being quarantined.

**Table 1:** Demographic characteristics of the participants (*N* = 300).

**Variables**	**N (%) or Mean ± SD**
Sex (male)	166 (55.3)
Public worker	
National government worker	180 (60.0)
Local government worker	120 (40.0)
Age	38.3 ± 9.1
18–29	61 (20.3)
30–39	105 (35.0)
40–49	88 (29.3)
50–60	46 (15.3)
Marital status	
Single	136 (45.3)
Married, without children	31 (10.3)
Married, with children	131 (43.7)
Other	2 (0.7)
Duration of employment (year)	10.3 ± 8.8
COVID-19 questions	143 (15.3)
Did you have experience addressing confirmed COVID-19 cases? (Yes)	83 (27.7)
Did you experience being quarantined due to being diagnosed with COVID-19? (Yes)	45 (15.0)
Did you experience being diagnosed with COVID-19? (Yes)	0 (0.0)
Psychiatric history	
Did you have experience in or treated depression, anxiety, or insomnia? (Yes)	46 (15.3)
Now, do you think you are depressed or anxious or do you need help for your mood state? (Yes)	37 (12.3)

### Applicability of Adapted SAVE-9 vs. SAVE-6 Scale Among Public Workers

#### (1) Adapted Version of the SAVE-9 Scale

The distribution of all nine items of the adapted SAVE-9 was within the normal limit based on the skewness and kurtosis for an acceptable limit range of ± 2 (Table 2). The KMO measure of the adapted SAVE-9 was 0.84, and Bartlett's test of sphericity had *p* value of < 0.001, which showed that these data were suitable for conducting factor analyses. The two-factor model of the adapted SAVE-9 scale was explored by PCA based on an eigenvalue plot (first factor eigenvalue = 4.105, second factor eigenvalue = 1.182) of Factor I—items 1, 2, 3, 4, 5, 7, 8, and 9 and Factor II—item 6. However, to determine the number of factors, results from the parallel analysis using MRFA extraction suggested the single structure model (real-data eigenvalue = 55.67, 95 percentile of random eigenvalue = 26.94). Therefore, we adopted the single-structure model of the adapted public workers' version of the SAVE-9 scale. The CFA of the adapted SAVE-9 did not reveal a good fit for the indices (CFI = 0.824, TLI = 0.765, RMSEA = 0.142, SRMR = 0.076). Therefore, we were not able to adopt the adapted version of the SAVE-9.

**Table 2:** Factor structure of the public workers' version of the SAVE-9 and factor loadings.

**Items**	**Responses (%)**					**Mean ± SD**	**Skewness**	**Kurtosis**	**Factor loading**	
	**0**	**1**	**2**	**3**	**4**				**Adapted SAVE-9**	**SAVE-6**
1. Are you afraid the virus outbreak will continue indefinitely?^*^	1.0	3.7	12.7	47.3	35.5	3.12 ± 0.84	−1.082	1.479	0.693	0.714
2. Are you afraid your health will worsen because of the virus?^*^	1.7	3.3	19.7	48.7	26.7	2.95 ± 0.86	−0.894	1.148	0.782	0.788
3. Are you worried that you might get infected?^*^	2.0	9.3	22.7	49.0	17.0	2.70 ± 0.93	−0.699	0.278	0.790	0.813
4. Are you more sensitive toward minor physical symptoms than usual?^*^	3.0	6.7	23.0	47.0	20.3	2.75 ± 0.95	−0.801	0.560	0.755	0.758
5. Are you worried that others might avoid you even after the infection risk has been minimized?^*^	7.0	28.0	25.7	26.3	13.0	2.10 ± 1.16	0.030	−0.946	0.605	0.602
6. Do you feel skeptical about your job after going through this experience?	14.3	29.3	25.0	23.3	8.0	1.81 ± 1.18	0.135	−0.928	0.505	–
7. After this experience, do you think you will avoid dealing with visitors with viral illnesses?	2.3	10.0	24.3	47.7	15.7	2.64 ± 0.94	−0.658	0.178	0.691	–
8. Do you worry your family or friends may become infected because of you?^*^	3.0	5.0	15.7	45.7	30.7	2.96 ± 0.97	−1.074	1.087	0.798	0.701
9. Do you think that your colleagues would have more work to do due to your absence from a possible quarantine and might blame you?	1.3	9.3	28.0	39.7	21.7	2.71 ± 0.95	−0.447	−0.259	0.693	–

* *Items of the Stress and Anxiety to Viral Epidemic-6 items scale*.

#### (2) SAVE-6 Scale

Similarly, the distribution of all six items of SAVE-6 was within the normal limit based on the skewness and kurtosis for an acceptable limit range of ± 2 (Table 2). The KMO measure of the SAVE-6 was 0.82, and Bartlett's test of sphericity showed a *p* value of < 0.001, which indicated that this data was suitable for conducting factor analyses. Parallel analysis using MRFA extraction indicated that use of the single structure model of the SAVE-6 (real-data eigenvalue = 71.18, 95 percentile of random eigenvalue = 34.47) was advisable. In addition, the CFA of the SAVE-6 showed a good model fit (CFI = 0.910, TLI = 0.850, RMSEA = 0.142, RSMR = 0.053). Therefore, we adopted SAVE-6 scale to assess the anxiety response of public workers to the viral epidemic. The multi-group CFA results showed that SAVE-6 measures the anxiety response of public workers the same way across genders (men vs. women, CFI = 0.918, TLI = 0.863, RMSEA = 0.135, SRMR = 0.057), types of public workers (national government worker vs. local government worker, CFI = 0.895, TLI = 0.825, RMSEA = 0.155, SRMR = 0.062), and the presence or absence of anxiety (GAD-7 ≥ 5 vs. GAD-7 < 5, CFI = 0.914, TLI = 0.857, RMSEA = 0.132, SRMR = 0.058).

### Reliability of the SAVE-6 Scores and Evidence Based on Relations to Other Variables

The SAVE-6 scale showed good internal consistency (Cronbach's alpha = 0.817 and McDonald's Omega = 0.818) and good convergent validity based on Spearman's correlation coefficient with GAD-7 (rho = 0.417, *p* < 0.001) and PHQ-9 (rho = 0.317, *p* < 0.001) scale scores. SAVE-6 scores were significantly higher for participants identified as having generalized anxiety symptoms [GAD-7 ≥ 10, t_(298)_ = 4.504, *p* < 0.001] and depression (PHQ-9 ≥ 10, [t_(298)_ = 4.063, *p* < 0.001)]. In addition, the SAVE-6 scale score was higher in females [vs. male, t_(298)_ = 2.756, *p* = 0.006] and in workers who are experienced in interacting with infected people [t_(297)_ = 3.208, *p* = 0.001] but not in older workers [junior 20–39 years old vs. senior over 40, t_(298)_ = 0.298, *p* = 0.853], workers in local governments [vs. the national government, t_(298)_ = 1.555, *p* = 0.121], and those who had experienced being quarantined [t_(298)_ = 0.483, *p* = 0.298)].

### Cut-off Score for SAVE-6 Among Public Workers

When we conducted the ROC analysis to determine the appropriate cut-off score of the SAVE-6 scale in accordance with the mild degree of generalized anxiety symptoms (GAD-7 score of 5), the appropriate cut-off point was calculated to be ≥ 16 (area under the curve [AUC] = 0.789, sensitivity = 0.81, specificity = 0.59).

## Discussion

In this study, we explored which of SAVE-9, adapted for public workers, and SAVE-6, derived for the general population, is more applicable to measure the anxiety symptoms of public workers specifically regarding the viral epidemic. We demonstrated that the SAVE-6 scale, as compared to the adapted SAVE-9 scale, was the more reliable and valid tool to assess the anxiety response of public workers specifically due to the COVID-19 pandemic. The SAVE-6 showed good internal consistency and good convergent validity with existing rating scales such as GAD-7 and PHQ-9 among a sample of public workers. Furthermore, the appropriate cut-off score for SAVE-6 was 16 in accordance with the mild degree of generalized anxiety symptoms based on the GAD-7 scale.

In this study we opted to apply the SAVE-6 scale, rather than the adapted version of the SAVE-9 scale, to assess public workers' anxiety symptoms specifically in response to the viral epidemic. The SAVE-6 scale has already been validated among the general population in South Korea (12) and Lebanon (16). In addition, we observed that the single-factor structure of SAVE-6 showed good internal consistency and convergent validity with other existing anxiety scales such as GAD-7 and coronavirus-specific anxiety rating scales, for example, the Coronavirus Anxiety Scale (28), among special populations such as medical students (17) or cancer patients (18). The results in this study added additional evidence that SAVE-6 can be reliably applied to assess the anxiety symptoms of public workers in response to the viral epidemic.

However, we were not able to adopt the single-structured model of the adapted version of the SAVE-9 scale with this sample. The original SAVE-9 scale, originally developed for healthcare workers, was observed to be clustered into two factors: the factor I, anxiety about the viral pandemic, and the factor II, work-related stress associated with the viral pandemic. We have already explored the applicability of factor I of the SAVE-9 scale, namely SAVE-6, for measuring the anxiety symptoms of the general population during this pandemic era. We first attempted to adapt the SAVE-9 scale for public workers, as public workers can play a similar role to that of healthcare workers during the COVID-19 pandemic. However, in this study, we observed that the adapted SAVE-9 scale cannot be reliably used to assess public workers' anxiety response to the viral epidemic, based on poor model fitness. First, it is likely that the factor II in the original SAVE-9 scale is more specific to healthcare workers rather than public workers. The specific encounter with infected cases, which public workers are experiencing, is not similar to that of healthcare workers. Instead of treating infected cases face-to-face, public workers are working on how to control the pandemic, save lives, ensure social protection, and sustain the economy (4). Even though they also work to keep people healthy, healthcare workers directly treat infected patients or patients with a higher risk of infectivity. This difference in the roles of public workers and healthcare workers may influence the difference in anxiety symptoms due to the viral pandemic. Second, the time frame when the survey was conducted in this study was different from that in the original SAVE-9 scale study. The original SAVE-9 survey was conducted one year ago (April 20–30th, 2020). It is likely that, this year, the participants have already adjusted to the new normal era and responded differently to the eight items in the original scale except for the revised item 7. Third, the sampling variability may have affected the difference in the factor structure. It may depend on the participant's role and work environment.

In this sample, the SAVE-6 scale score was higher for females and workers who are experienced in addressing infected people. We cannot directly compare the results with previous studies because there are few studies on the mental health of public workers. Previous studies have shown that, among healthcare workers, females, nurses, and workers dealing with patients infected with COVID-19 are more likely to experience stress and anxiety when measured with a scale used for general stress and anxiety (9, 29), as opposed to scales specific to a viral epidemic. Similarly, in this study, female public workers and those who participated in interaction with infected people were shown to experience greater stress. The present results also correspond to those from our earlier study on healthcare workers (30). Use of a scale on general stress and anxiety entails a limitation in pinpointing COVID-19-related stress and anxiety. Instead, a scale specialized for a viral epidemic will allow for evaluation of stress and anxiety that stems from the present pandemic more accurately and help establish a support system for public workers.

In this study, the appropriate cut-off score of the SAVE-6 scale was defined as 16 in accordance with the mild degree of GAD-7 scale (point of 5). Similarly, we reported a point of 15 as a cut-off value for factor I (SAVE-6) among healthcare workers (11) and medical students (17). However, we observed a relatively lower cut-off of 12 among the Lebanese general population (16). We should thus consider that a cut-off score can vary depending on the samples, ethnicity, and gender. Furthermore, the present cut-off score was explored by reflecting on the situation one year after the first wave of COVID-19. Because the time frame and targeted population might affect the cut-off value, further studies using additional various samples are needed.

This study had several limitations. First, due to the pandemic, the current study had to be conducted via an online survey rather than interviewing participants face-to-face in a more structured way. Under this way, only those who were enrolled in the survey website could participate in the present study, possibly leading to biased data. However, we conducted this study via the online survey to gather responses from various cities in South Korea without face-to-face contact with the participants in this pandemic era. Second, there were no confirmed COVID-19 patients in the study sample. The results may have been different if participants who experienced being infected had been included. Previously, a high level of anxiety symptoms was observed among individuals with COVID-19 (31). As more participants became infected, the mean level of generalized anxiety increased. Though we cannot predict precisely, a higher proportion of anxious population may increase the sensitivity of the SAVE-6 scale and, consequently, decrease the cut-off score. Third, the role of public workers in this sample was not specifically described. The results can vary depending on the specific role of the public workers. The adapted version of the SAVE-9 might be applicable to public workers who are directly involved in COVID-19-related fields and perform stressful tasks, as the original SAVE-9 scale was developed for frontline healthcare workers. Therefore, it was important to describe the work role of participants in detail. However, in this study, we observed that the SAVE-6 scale, which was developed for the general population, as compared to the adapted version of the SAVE-9 scale, was more reliable for and applicable to public workers.

In conclusion, we observed that the SAVE-6 scale, as opposed to the adapted version of the SAVE-9 scale, can be applied with good reliability and validity to assess the anxiety symptoms of public workers specifically in response to the viral epidemic.

## Data Availability Statement

The raw data supporting the conclusions of this article will be made available by the authors, without undue reservation.

## Ethics Statement

The studies involving human participants were reviewed and approved by Institutional Review Board of Asan Medical Center. Written informed consent for participation was not required for this study in accordance with the national legislation and the institutional requirements.

## Author Contributions

SC contributed to conception and design of the study and performed the statistical analysis. CP, GJ, and SC wrote the first draft of the manuscript. KY, SL, and SS wrote sections of the manuscript. All authors contributed to manuscript revision, read, and approved the submitted version.

## Funding

This work was supported under the Framework of International Cooperation Program managed by the National Research Foundation of Korea (FY2020K2A9A1A01094956). The funding source was not involved in the study design; in the collection, analysis, or interpretation of data; in the writing of the report; or in the decision to submit the article for publication.

## Conflict of Interest

The authors declare that the research was conducted in the absence of any commercial or financial relationships that could be construed as a potential conflict of interest.

## Publisher's Note

All claims expressed in this article are solely those of the authors and do not necessarily represent those of their affiliated organizations, or those of the publisher, the editors and the reviewers. Any product that may be evaluated in this article, or claim that may be made by its manufacturer, is not guaranteed or endorsed by the publisher.

## Footnotes

^1^www.embrain.com.

^2^www.save-viralepidemic.net.
